# Differential root zone secretions and the role of root border cells in rhizosphere manipulation

**DOI:** 10.1007/s11101-025-10084-y

**Published:** 2025-02-19

**Authors:** Clayton Kranawetter, Lloyd W. Sumner

**Affiliations:** 1 Department of Biochemistry, University of Missouri-Columbia, Columbia, Missouri 65211 USA; 2 MU Metabolomics Center, University of Missouri-Columbia, Columbia, Missouri 65211 USA; 3 Christopher S. Bond Life Sciences Center, University of Missouri-Columbia, Columbia, Missouri 65211 USA; 4 Interdisciplinary Plant Group (IPG), University of Missouri-Columbia, Columbia, Missouri 65211 USA

**Keywords:** Root border cells, Rhizosphere metabolites, Root secretions, Plant–microbe interactions

## Abstract

Root tissues are broadly divided into mature tissue, elongation zone (developing tissue), root tip, and border cells. While each zone contributes individually to the overall root secretion profile, border cells are emphasized in this review due to their specialized secretory functions. Border cells are often overlooked in plant root focused studies, thus excluding an important component of root functionality. Border cells are a specialized cell type surrounding the root apical meristematic region of most plant species, with the exception of the *Brassicaceae* family that possess border-like cells. Both cell types share the commonality of complete detachment from the root tip and reliance on internal starch reserves to perform metabolic processes. However, border cells release from the root tip as single/individual cells whereas border-like cells separate as cohesive sheets. Furthermore, border cells, but not border-like cells, secrete a complex matrix consisting of mucilage, proteins, DNA, and metabolites. Many of these secreted compounds are bioactive (e.g. secreted mucilage supports microbial growth and DNA physically entangles pathogens) thus mediating symbiosis and pathogen defense. We are interested in metabolites secreted from individual root regions, with a heavy emphasis on those specifically arising from border cells. Border cell metabolite secretion is in need of further investigation, as current research indicates they secrete symbiosis-inducing, allelopathic, and defense oriented metabolites. This review will summarize current literature regarding metabolite secretions by specific root cell types and regions. In particular, it will focus on border cell contributions to the rhizosphere chemistry relative to other root tissue types.

**Graphical abstract:**

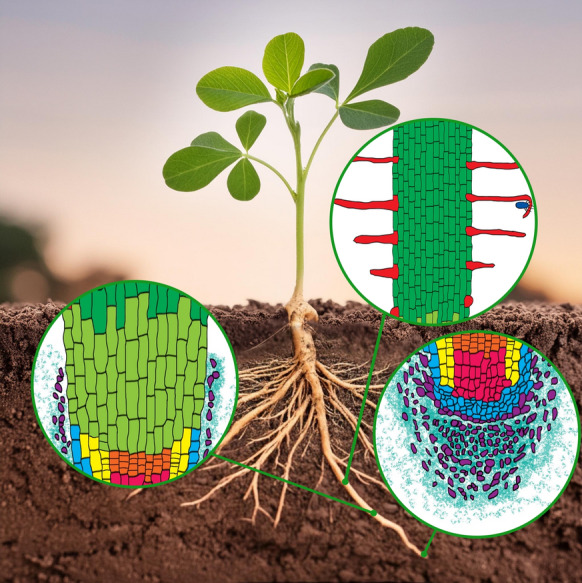

## Introduction

The goal of this document is to review current literature concerning root-based metabolite secretions from discrete tissue regions, with a focus on metabolite identities and methods of secretion. In particular, this review focuses on the origin of secreted compounds from mature plant root tissues (i.e. fully developed tissues), elongation zone (developing tissues), meristem/columella/peripheral cells (broadly defined as the root tip), and border cells.

Root border cells are major contributors to the overall root secretory profile, making them an important, yet often overlooked, root tissue type. Current research shows that individual root tissues perform discrete functions that cannot be generalized or combined in an attempt at simplicity (Birnbaum et al. 2003; Moussaieff et al. 2013). Individual root tissues work harmoniously together, with some regions such as the root tip and border cells engaging heavily in secretion while other areas, such as the elongation zone and mature tissue, focusing more on nutrient utilization rather than rhizosphere secretions. Understanding how each region works individually and in concert will help further understanding of the dynamics behind nutrient acquisition, rhizosphere recruitment/assembly, and patterns of pathogenesis. With recent evidence that differential microbial colonization occurs along the root axis, the role of individual root regions in secretion and rhizosphere interactions represents a major knowledge gap in need of deeper study (Loo et al. 2024).

Metabolite analyses of individual root regions are challenging due to accurate tissue harvest/separation that minimizes metabolic disruptions, limited quantities available for analyses, and availability of highly sensitive instrumentation (for mass spectrometry and deeper genomics/transcriptomics investigations). Border cells, in particular, remain under-studied largely due to the necessity for a thoughtful approach in plant growth and tissue harvest (i.e. border cell dispersal in water) and limiting tissue amounts. However, the pace of technological development has drastically reduced this challenge, especially with improvements in mass spectrometry imaging (including Desorption ElectroSpray Ionization/DESI and Matrix-Assisted Laser Desorption Ionization/MALDI imaging), increased sensitivity of newer instruments, and availability of single cell-oriented mass spectrometers. With the ever-increasing availability and affordability of this highly sensitive equipment and lower barrier to tissue harvest, it is time to re-examine our approach to holistic root biology.

This review provides detailed coverage of border cell production mechanisms, border cell secretions, and the role(s) of these secretions in mediation of plant–microbe interactions. The metabolite secretions of each root tissue type will be divided into primary (e.g. organic acids, sugars, and amino acids) and specialized metabolites. Each of these sections will be further divided in two ways. The first will be secretion activities based upon discrete root tissue types/regions (i.e. border cells, root tip, elongation zone, or mature tissues). The second will be different secretion mechanisms/transporters as known and/or appropriate with a focus on metabolites discussed in this review.

### Border cell formation

**Fig. 1:**
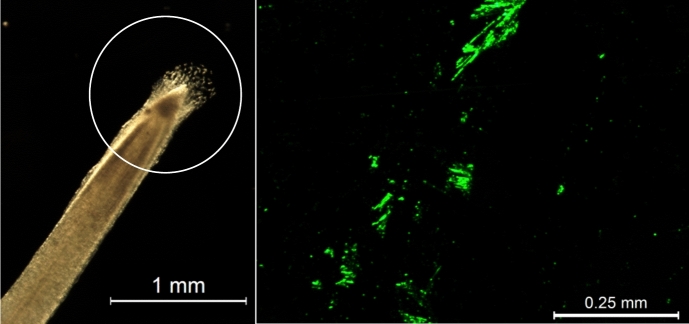
Visualization of *M. truncatula* Border Cells. (Left, White Circle). Border cells surround the apical meristem of many plant species and disperse upon exposure to water. (Right) Fluorescein diacetate cell viability stain indicates border cells remain alive even after their removal from the root and appear as clumps of cells

Border cells are a specialized cell/tissue type surrounding the apical meristematic region of many agronomically important plant species, such as *Zea mays* (corn), *Glycine max* (soybean), *Medicago sativa* (Alfalfa), *Oryza sativa* (Rice), and *Pisum sativum* (Pea) (Hawes and Pueppke 1986). Unlike other plant tissues, border cells are not physically connected to the root tip in any way. Instead, they are held in place through a complex, self-secreted, water-soluble matrix consisting of mucilage, DNA, proteins, and metabolites (Wen et al. 2007, 2009; Watson et al. 2015). The presence of water results in near-immediate dispersal of border cells (Fig. 1) (Hawes and Pueppke 1986). After removal, a high percentage of border cells in water are alive (>90 percent) and a small percentage of border cells (6 percent in *Pisum sativum* and 10 percent in *Glycine max*) remain viable up to 10 days in water alone (Hawes and Pueppke 1986). Increased viability is seen (51 percent in *Pisum sativum* and 60 in *Glycine max*) at 22 days in RM medium (Hawes and Pueppke 1986). The highest percentage and longest survival rate of border cells removed from the root has been observed in RM medium supplemented with 1.1 mg of 2,4-D and coconut milk, resulting in 95% survival of *Pisum sativum* and 96% in *Glycine max* at 31 days (Hawes and Pueppke 1986).

Cells at the plant root tip utilize phloem, symplastic (direct cell-to-cell through plasmodesmata) and apoplastic (exiting one cell entirely to enter another cell) sucrose from source tissues for energy and starch accumulation (Hennion et al. 2019). Cells behind the meristem in the elongation zone use this starch for continued growth and development. Columella cells in front of the root meristem use the starch granules for gravity sensing (Blancaflor et al. 1998). Border cells rely on internal starch granules for energy once detached from the root, breaking it down to support cellular processes in the absence of fixed carbon provided through photosynthetic activities (Watson et al. 2015). Visual comparison of border cell starch granules to those of other root tip tissues revealed comparatively smaller granules, indicating increased utilization in border cells (Watson et al. 2015). Deeper experimentation using qRT-PCR revealed a 15 × increase in β-amylase (an enzyme that breaks down starch) in border cell transcripts compared to the root tip, providing biochemical support to visual comparison of starch granules (Watson et al. 2015).

Removal of border cells from the root activates mitosis in the root meristem, resulting in the production of new cells that progress/differentiate outward from the root meristem as columella cells, peripheral cells, and lastly epidermal cells (Brigham et al. 1998). Upon reaching the outer layer of the root tip epidermis, pectin methylesterase activity increases, leading to methylation and breakdown of the pectin making up plant root cell walls (Hawes and Lin 1990; Stephenson and Hawes 1994; Brigham et al. 1998). Increased pectin methylesterase activity is coordinated with border cell removal, as *Pisum sativum RCPME1* (a pectin methylesterase associated gene) mRNA expression increases within 5 min and pectin methylesterase enzymatic activity increases within 2.5 h of border cell removal (Stephenson and Hawes 1994; Wen et al. 1999). At this point, single or small groups of cells physically detach from the root tip, differentiating into border cells (Watson et al. 2015). Based on these results, pectin methylesterase activity is essential for border cell production. Indeed, partial disruption of pectin methylesterase translation using antisense mRNA targeting *RCPME1* in hairy root cultures stunted root growth in the elongation zone and produced border cells that did not detach from the root tip (Wen et al. 1999).

Upon reaching a species-specific number of border cells (e.g. *Medicago truncatula* possesses approximately 3000–4000 border cells per root whereas corn (*Zea mays* L. “W64”) possesses approximately 2000–3000), root meristem mitotic activity is reduced and border cell production ceases until removal occurs again (Hawes and Pueppke 1986; Brigham et al. 1998; Watson et al. 2015). While individual plant species demonstrate a predetermined range of border cell numbers, environmental conditions can modify the total number of border cells. For example, exposure of pea (*Pisum sativum*) roots to 50 µM pea phytoalexin pisatin increased total root border cell numbers to 7767 ± 345 compared to 3878 ± 387 border cells at 1 µM pisatin and 3988 ± 420 with no applied pisatin (Curlango-Rivera et al. 2010). In contrast, application of apigenin at the same range of concentrations had no effect on total border cell numbers (Curlango-Rivera et al. 2010). Furthermore, high concentrations of CO_2_ stimulated an increase in border cells in pea, but not alfalfa (*Medicago sativa*) under the same conditions, indicating a possible species-specific response in border cell production to environmental conditions (Zhao et al. 2000a).

#### Border-like cells are only seen in the *Brassicaceae* family and distinct from border cells

While border cell production can be generalized to many plant species, it is worth mentioning that the Brassicaceae family, in which the popular plant model system *Arabidopsis thaliana* resides, possess a similar tissue type termed border-like cells. Distinguishment of border-like cells from border cells is imperative, as border-like cells possess a distinct physiology and, thus far, have proven to possess a subset of traditional border cell secretory functions. Foremost, border-like cells separate from root tips as cohesive sheets of cells, compared to border cells which separate as individual cells or small groups (Driouich et al. 2007; Hawes et al. 2016b). Interestingly, this physiological difference seems to be partly attributable to NIN-LIKE PROTEIN7 (NLP7) in *Arabidopsis thaliana*, mutation of which alters border-like cell production from sheets to single/small groups of cells more like traditional border cells (Karve et al. 2016). That being said, this review will limit discussions of *Arabidopsis thaliana* border-like cells as these cells are physiologically different from and have not yet been demonstrated to possess the same secretion functions as border cells. If necessary, all information arising from *Arabidopsis thaliana*-based research will be appropriately designated such that it can be evaluated with discretion.

### Border cells differentiate after root separation and secrete numerous materials

There are large scale changes in border cell gene expression, protein translation, and metabolite accumulation after separation from the root (Brigham et al. 1995; Watson et al. 2015; Kranawetter et al. 2021). Upon release, border cells secrete mucilage, DNA (by a currently unknown mechanism), proteins, and metabolites (Wen et al. 2007, 2009; Watson et al. 2015; Kranawetter et al. 2021). Effectively, border cells are differentiating from progenitor tissues, making border cells a distinct tissue type. The transcriptional changes that occur redirect carbon resources (using starch granules for energy) to specialized metabolism, with increased transcripts associated with phenylpropanoid, lignin/lignan, flavonoid, isoflavonoid, and terpenoid biosynthesis (Watson et al. 2015). In coordination with these transcriptional changes, the translational/protein profile both within and secreted by border cells differs from the root tip (Brigham et al. 1995; Wen et al. 2007). Of newly produced border cell proteins, approximately 25% are secreted within one hour of border cell isolation (Brigham et al. 1995). The transition to new protein production is rapid, with border cells incorporating [^35^S] labeled cysteine and methionine 2.6-fold faster than root tip cells (Brigham et al. 1995). These secreted proteins are essential in defense of the root tip, as infection of pea root tips by *Nectria haematococca* (a fungal pathogen) increased after prevention of border cell protein secretion using Brefeldin A and proteolytic degradation of secreted proteins using proteinase K (Wen et al. 2007). Some of the 32 border cell secreted proteins have known defense functions, among them being histone H4 bound to secreted DNA (Wen et al. 2007; Hawes et al. 2012). A related Histone H5 has established antimicrobial properties, although the mechanism behind this remains unknown it has been proposed to be related to histone structural domains (α-helices) (Jodoin and Hincke 2018). Border cells from transgenic Bt-Cotton plants secrete Cry1Ac and Cry2Ab proteins, which are potent antimicrobial compounds, indicating possible usage in precision agriculture (Knox et al. 2007).

#### Border cell secreted mucilage in abiotic and biotic responses

It has been reported multiple times that root border cells contribute to the secretion of mucilage (Miyasaka and Hawes 2001; Huskey et al. 2018). The carbohydrate composition of this mucilage varies between plant species (Knee et al. 2001). For example, Pea mucilage was found to contain 33.5% arabinose whereas Maize mucilage was found to contain 16% arabinose (Knee et al. 2001). Furthermore, mucilage from *M. truncatula* mainly consists of xylogalacturonan, underscoring the differences in mucilage carbohydrate composition depending on plant species (Wang et al. 2017). Mucilage is produced even after border cell removal from the root, indicating that it can be produced independently of any materials possibly provided by the root tip (Miyasaka and Hawes 2001; Hawes et al. 2016b; Huskey et al. 2018). Secreted mucilage likely helps lubricate root movement through the soil while aiding in retainment of border cells at the root tip. The biological basis underlying differential root exudate carbohydrate composition depending on plant species remains unknown. However, it is clearly an area worthy of further study in fundamental plant–microbe rhizosphere interactions.

Mucilage secretion has been associated with resistance to toxic levels of soil aluminum (Hawes et al. 2016b). Aluminum buildup in soils can lead to cessation of cell elongation and eventual death of the plant (Jones 1998a). Aluminum resistant strains of plants have been shown to have increased mucilage secretion relative to less resistant phenotypes, hinting at role for secreted mucilage as an aluminum “detoxifier” (Miyasaka and Hawes 2001). Mucilage secretion as a detoxification (or trapping) mechanism also appears to be possible for other toxic metals. Lead, like aluminum, damages the apical meristem of roots and ultimately inhibits root growth (Huskey et al. 2018, 2019). Border cell secreted mucilage accumulates lead, thereby trapping it and preventing root meristem exposure (Huskey et al. 2018, 2019). This mechanism of preventing root tip exposure seems to extend beyond toxic metals, as secreted mucilage also plays a role in drought resistance, either through retainment of root moisture (i.e. keeping the root tip from drying out) or absorbing low levels of moisture remaining in the soil (i.e. drawing in water from the soil to the root) (Ahmed et al. 2014). Secreted mucilage also lubricates root movement through the soil, allowing continued movement even under dry conditions. This allows roots to penetrate further into the soil and reach distant water reservoirs.

The diversity of secreted mucilage functions is not limited to abiotic interactions but is also associated with biotic interactions. Isolated mucilage from pea supported growth of *Rhizobium leguminosarum*, serving as a primary carbon source (Knee et al. 2001). As the sugar constituents of mucilage are chemoattractants, and soil microbes likely possess some preference in carbohydrates, there is likely an interesting species-specific rhizosphere crosstalk occurring (Knee et al. 2001). Effectively, this could translate to a given plant species secreting mucilage composed of specific sugar constituents to attract specific microbial species. With border cell mucilage and arabinogalactan protein secretion, there is likely a complex interplay aiding mutualistic microbial species root attachment and pathogen defenses (Allan Downie and Allan Downie 2010; Xie et al. 2012; Driouich et al. 2013).

It is well known that roots secrete mucilage, with the typical site of secretion being generalized to the root cap region. Further investigation into the site(s) of mucilage secretion demonstrated that not all root cap tissues are responsible for mucilage secretion (Wang et al. 2017). Root border and peripheral cells have more *trans*-Golgi cisternae and are more vacuolated than meristematic and columella cells, possessing large vesicles that contain mucilage cargo (Wang et al. 2017). While these large vesicles are also present in peripheral root cells, they only appear to fuse with the plasma membrane in border cells (Wang et al. 2017). In *Medicago truncatula*, it appears that mucilage is packaged in the early trans-Golgi cisternae, budding off into large vesicles (Wang et al. 2017). These vesicles then travel to, and fuse with, the plasma membrane, depositing their mucilage cargo into the rhizosphere (Wang et al. 2017). While it is generalized that the root cap produces mucilage, the data covered above presents compelling evidence that this is not the case. Such generalizations must be approached cautiously or even discouraged, as border and perhaps peripheral cells appear to be the primary source of secreted mucilage unlike other root cap tissues.

#### Border cell secreted DNA is essential in root defense

DNA secretion is another rather fascinating aspect of border cell functions, as it is indispensable in defense against soil pathogens (Wen et al. 2009; Hawes et al. 2011). This secreted DNA is remarkable in its functional similarities to mammalian neutrophil extracellular traps (NETs) (Brinkmann et al. 2004; Hawes et al. 2015). Extracellular DNA functions as a physical barrier to presumably entangle pathogenic microbes and prevent their motility (Hawes et al. 2016a). Enzymatic removal of secreted DNA using deoxyribonuclease (DNase) abolishes defense against pathogenic microbes, allowing infection to proceed (Hawes et al. 2016a). Some pathogenic microbes (such as *Ralstonia solanacearum*) have evolved the ability to secrete DNases that enable escape from secreted DNA nets (Tran et al. 2016; Park et al. 2019). While unproven, the authors hypothesize degraded border cell secreted DNA (in the form of free nucleotides or short DNA fragments as a result of pathogenic secreted DNase activity) in the soil environment could lead to defense related cross communication and/or signaling mechanisms, serving as a Damage Associated Molecular Pattern (DAMP) (Boller and Felix 2009). In short, DAMP perception in plants initiates defense responses, aiding in prevention of pathogen infection (Boller and Felix 2009). Support for this hypothesis has been shown through DNA fragments (under 700 bp) derived from *Phaseolus vulgaris* applied to living *P. vulgaris* plants eliciting defense signaling processes (H_2_O_2_ production and MAP kinase activation) and decreased *Pseudomonas syringae* (a bacterial pathogen) infection (Duran-Flores and Heil 2018). This was shown to be species specific, as application of non-self DNA (i.e. non-*P. vulgaris* DNA) from *Phaseolus lunatus* or *Acacia farnesiana* induced lower H_2_O_2_ production compared to self-derived DNA (Duran-Flores and Heil 2018). Short border cell secreted DNA fragments produced via soil pathogen extracellular nuclease activity would likely have similar defense activation properties. Furthermore, extracellular ATP arising from degraded border cell secreted DNA could initiate defense responses in the host plant or neighboring plant roots. Extracellular ATP is known to initiate purinergic signaling and plant defense responses as a DAMP, including increases in cytosolic Ca^2+^ concentration, production of Reactive Oxygen Species and nitric oxide, activation of Mitogen Activated Protein (MAP) kinase cascade, and changes in metabolism (Tanaka et al. 2010; Choi et al. 2014; Cho et al. 2022). Essentially, border cell secreted DNA could function as a physical barrier to rhizosphere microbes and, as a result of pathogen secreted DNase activity, a preformed messenger (or a sort of early indicator of danger) of pathogen presence (Tripathi et al. 2018; Cho et al. 2022).

#### Border cell secretion of metabolites

Isolation and examination of border cell secreted metabolites is a subject of ongoing work, and information on this topic is limited. Current studies examining secreted metabolites were done in the context of total root secretions. Border cells in these experiments were isolated for microarray analysis of upregulated transcripts, qRT-PCR probing specific genes associated with metabolite biosynthesis, and metabolomics experiments (Watson et al. 2015; Kranawetter et al. 2021). This methodology enabled development of hypotheses targeting metabolites that could be secreted by border cells. Many root secreted metabolites also have known associations with defense, mediation of beneficial plant–microbe relationships, and acquisition of nutrients (Watson et al. 2015; Kranawetter et al. 2021). However, these studies do not establish the specific secretions of isolated border cells. At a minimum, some of these metabolites are arising from border cells, and these metabolite secretions will be discussed throughout this document. Current work is investigating border cell specific secreted metabolites, aiming to fill this knowledge gap (Kranawetter et al. 2021).

### Border cell involvement in rhizosphere dynamics

**Fig. 2:**
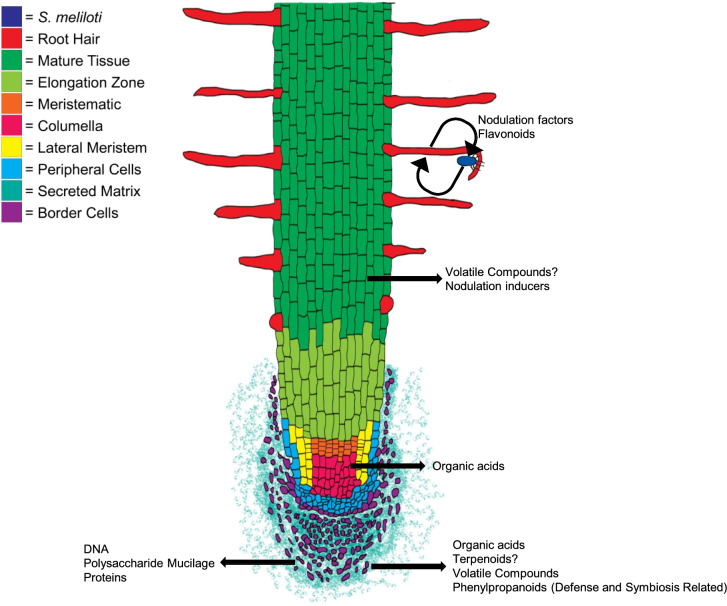
Summary of Secretions by Root Tissue Type. Broadly categorized differentiated tissue types are grouped by color. Individual tissue types perform discrete functions, with their secretions engaged in discrete root-rhizosphere dynamics. Border cells release diverse materials into the rhizosphere, reflecting various roles as rhizosphere modulators. Other regions of the root release compounds that trend towards nutrient acquisition and specialized plant–microbe or plant-plant interactions

A proper understanding of tissue specific root secretions is necessary for proper attribution to overall root function and activity. As plant roots engage in complex, multi-organismal activities, it is necessary to understand what specific root tissues are facilitating these activities. A generalization does not suffice, as it is becoming ever more essential to understand how roots are directly mediating plant–microbe and plant-soil interactions. With the knowledge that border cells are major secretory machines (i.e. releasing mucilage, proteins, DNA, and metabolites) the origin of soil metabolites becomes complicated (Wen et al. 2007, 2017; Watson et al. 2015; Wang et al. 2017; Huskey et al. 2018) (Fig. 2). There is evidence indicating some level of metabolite secretion originates from root hairs in the mature tissue region (Ahmad et al. 2021) (Fig. 2). The biological implications behind multiple sources of secretions remains unknown, but they likely relate to the discrete functions of differentiated tissue types. Many studies involving root dynamics do not address the presence of multiple secretion sources. Furthermore, border cells are often not included in studies focused on root-rhizosphere dynamics.

Some aspects of rhizosphere secretion/deposition of metabolites appear to be facilitated via diffusion, with others being active transport (Canarini et al. 2019). Rhizosphere deposition via facilitated diffusion or active transport seems to be true for both biotic and abiotic stress (Canarini et al. 2019). Naturally, some rhizosphere deposition contributions are also through normal cell lysis/death. Among these direct and indirect manipulations of the rhizosphere, there is consistent data showing robust defense against pathogenic microbes. However, there is great complexity in root secretion-based manipulation of rhizosphere microbial communities. An overarching biological theme behind root secretions is rhizosphere manipulation to create favorable conditions for plant health and growth. There is extensive cross-talk between the rhizosphere and plant roots, with root exudates modulating soil microbial communities and microbial communities altering root growth (Vacheron et al. 2013; Hu et al. 2018; Korenblum et al. 2020). Border cells play at least some role in this manipulation, as colonization by arbuscular mycorrhizae is positively correlated with increases in border cell numbers in the Amaranthacea family (*Amaranthus tricolor*, *Celsia cristata*, *Gomphrena globosa*, and *Amaranthus caudatus*) along with *Trifolium repens* (Arriola et al. 1997).

## Root secretions and their roles in rhizosphere interactions

Root secretions are exceptionally diverse, encompassing primary metabolites (such as amino acids, organic acids, and fatty acids) and specialized metabolites (such as phenolics, saponins, sapogenins, and glucosinolates) (Jones et al. 2009). This section begins with primary metabolite secretions and then moves into specialized metabolites. Within these sections, we will discuss metabolite identities, functions, secretion processes, and root tissue origin.

### Primary metabolites in nutrient acquisition and rhizosphere modification

#### Organic acids

Organic acids, which are foundational in plant nutrient acquisition and serve as primary carbon sources for rhizosphere microbes, are omnipresent throughout all plant species. This compound class serves as essential, CHO (Carbon, Hydrogen, Oxygen) containing, low molecular weight compounds involved in a variety of metabolic processes such as energy generation in the citric acid cycle. The ability of plants to secrete organic acids enables direct, dynamic manipulation of local rhizosphere conditions, aiding in damage mitigation, drought tolerance, or nutrient acquisition (Jones 1998a; Canarini et al. 2019). Such manipulations can be in response to abiotic stresses (i.e. nutrient deficiencies, salt stresses, drought, etc.) and organic acids, which possess varying levels of negative charge, can help mobilize various soil bound metals (Jones 1998a). For example, malate secretion is increased under toxic aluminum exposure or phosphorous deficiency, presumably to mobilize these metals for detoxification and/or uptake respectively (Canarini et al. 2019). The binding potential of organic acids to soil bound metals, such as iron, phosphorous, and magnesium, allows complexation of insoluble versions of soil bound metals (such as Fe^3+^) for charge reduction to a soluble complexed form suitable for root absorption (such as Fe^2+^). Synthesis of malate, lactate, fumarate, and aspartate are increased in response to drought stress, presumably functioning as osmolytes and aiding in water retention (Gargallo-Garriga et al. 2018).

The sites of organic acid secretion remain complex, with secretion possibly arising from multiple sources. Border cells possess relatively high levels of multiple organic acids, (as measured by mass spectrometry), such as citric, malic, and succinic acid among several others relative to the root tip (Watson et al. 2015; Kranawetter et al. 2021). Border cells demonstrate an increase in citric acid cycle intermediates after separation from the root (Watson et al. 2015). The genetic expression within border cells also changes after separation, diverting cellular functions towards enzymatic machinery necessary for organic acid synthesis (Watson et al. 2015). While such enzyme-associated transcripts do not provide direct evidence of increased organic acid synthesis and secretion, it is feasible that these organic acids are deposited in the rhizosphere. The capabilities of border cells in organic acid secretion remains a subject of ongoing research. Other areas of the root likely contribute to the overall rhizosphere organic acid content as well. The base and tip of plant roots secrete malate and citrate, with varying levels of secretion based on nutrient sufficient/deficient status (Jones 1998a). Studies simplifying the root tip, rather than differentiating the root cap and border cells should be subjected to more critical speculation, as there are several different tissue phenotypes in the traditional “root tip”. The developed and developing tissues, when dissected, demonstrate differential localization of organic acids (Kranawetter et al. 2021). Mature tissue regions appear to release some levels of malate and citrate (Jones 1998a). Release of organic acids from discrete tissue regions is logical. As the root progresses through the soil, the apex regions (peripheral or border cells) secrete organic acids and protons that aid in mobilization of soil-bound metals. As the root tip moves further into the soil, these mobilized metals are available for uptake by mature/maturing tissues (Miguel et al. 2015). Supporting this model, the apex region is notably absent in iron transport machinery, with membrane iron transporters localizing in mature tissue regions (Jones 1998a).

#### Amino acids

Multiple amino acids have been reported in rhizosphere secretions, including tryptophan, phenylalanine, asparagine, valine, aspartate, and leucine (Kranawetter et al. 2021). Current research proposes that most amino acids are passively lost at the meristematic region (Jones et al. 2009; Canarini et al. 2019). However, many early studies did not separate border cells, possibly misattributing the meristematic region secretions. While this may seem innocuous, understanding that border cells are responsible for amino acid (and other compound) secretory functions is important for future secretion-based targeted analyses and manipulations. This would include metabolic engineering approaches, ensuring that border cells are manipulated rather than other tissues that would be otherwise unresponsive.

Some older reports found valine, phenylalanine, and leucine arising from root hair zone (which is an ambiguous term and could possibly mean the mature tissue or root hairs) (Badri and Vivanco 2009). However, these data should be examined with discretion due to the current availability (and previous lack) of sophisticated instrumentation capable of examining zonal secretions. Newer mass spectrometry-based evidence using Gas Chromatography coupled to Mass Spectrometry (GC–MS) indicates some amino acids are differentially accumulated or depleted in root hairs compared to stripped roots (Brechenmacher et al. 2010). With more evidence coupling amino acid membrane transporters in root hairs with mass spectrometry (as in Brechenmacher et al.), this could provide a clearer picture of root hair amino acid secretion. An ideal approach for demonstrating root hair secretion would utilize instrumentation capable of analyzing single cells as mentioned earlier in this document. While sensitivity challenges remain with single cell proteomics and metabolomics, continuing developments in mass spectrometry technologies have brought success within reach.

Secretion of phenylalanine could be from border cells, as enzyme associated transcripts along the phenylalanine biosynthetic pathway are upregulated in border cells compared to the root tip (Watson et al. 2015). It is also plausible that border cells are the main contributors of tryptophan to rhizosphere secretions, as a higher relative intensity of tryptophan and increased enzyme associated transcripts along the tryptophan biosynthetic pathway have been seen in border cells compared to the root tip (Watson et al. 2015; Kranawetter et al. 2021).

#### Sugars

Firm proof of carbohydrate secretion by specific root tissues is an area of ongoing research. There is a general consensus that the root tip is a major site of sugar secretion (Kuzyakov and Jones 2006; Jones et al. 2009). The composition of secreted sugars is varied and composed of glucose, sucrose, fructose, galactose, mannose, xylose, ribose, and arabinose among others (Shay and Hale 1973; Chen et al. 2015). Secretion of sugars, and the balance in secretion/uptake, into the rhizosphere plays an important role in plant–microbe interactions through establishment of both symbiotic and pathogenic interactions (Gabriel-Neumann et al. 2011; Chen et al. 2015; Jeena et al. 2019; Kim et al. 2021). For example, plant roots are known to release sucrose, which plays a role in the establishment of arbuscular mycorrhizal relationships in *Medicago truncatula*, tomato, and potato plants (Chen et al. 2015). Conversely, it has been shown that the Sugars Will be Eventually Effluxed Transporter 2 (SWEET2) in *Arabidopsis* is associated with glucose uptake and prevention of pathogen proliferation (Chen et al. 2015). Recent evidence has further shown in *Arabidopsis* that SWEETs accumulation along the root axis changes in response to the absence or presence of sucrose and microbial inoculants (Loo et al. 2024).

For legume species, there is indication of sugar transport in root nodules (Kryvoruchko et al. 2016; Sugiyama et al. 2017). However, whether these transporters are associated with rhizosphere secretion of sugars needs further study. From the perspective of sugar uptake in *Arabidopsis,* the transporter SWEET2 localizes in mature tissue, root cap, and root tip regions, providing potential indication of sugar secretion activities as well (Chen et al. 2015). There is no current evidence that border cells are involved in sucrose secretion. However, large quantities of polysaccharide mucilage (discussed earlier in this review) are secreted by border cells, and it is certainly possible other forms of sugars are being released to the rhizosphere (Knee et al. 2001; Wang et al. 2017). This is supported by upregulation of transcripts associated with starch breakdown/sucrose production in border cells (Watson et al. 2015). This includes a 15 × increase in β-amylase transcripts (which break down starch granules) and associated higher levels of sugars fructose, glucose, galactose, sucrose, and arabinose (Watson et al. 2015). Of these sugars, only sucrose was significantly higher compared to root tips, but the other sugar products remain credible leads for future studies (Watson et al. 2015). While this evidence is not proof of carbohydrate secretion (mono- or disaccharide units), the evidence of enhanced starch breakdown and known border cell secretion activities provide a reasonable biological basis for investigation of border cell carbohydrate secretion.

#### Secretion targets in future spatial secretion studies

The rest of this section will examine known secretion methods of organic acids, amino acids, and carbohydrates. Not all secretion mechanisms for these metabolites are known, and this section is designed to provide an overview of reported transport processes. This is not a comprehensive review of all transport methods, and we would direct readers to citations utilized in this section.

#### Passive transport

Mechanistically, organic acids/amino acids/sugars can leach across the lipid bilayer by means of an electrochemical or concentration gradient in conjunction with nonspecific transporters (such as a pore or channel) (Jones 1998a; Lee et al. 2007). This seems plausible as the surrounding soil would be expected to possess a lower organic acid concentration. Supporting this model is the evidence indicating root infections tend to reside in the elongation zone where secondary cell wall formation is incomplete, which provides easier infiltration routes (Gunawardena and Hawes 2002; Gunawardena et al. 2005). A lower rhizosphere concentration of organic acids, amino acids, and carbohydrates would result in movement of these compounds from the phloem offloading site to the rhizosphere through diffusion facilitated by passive transporters at the plasma membrane (Jones 1998a; Canarini et al. 2019). Some transporters have been established, such as Sugars Will be Eventually Effluxed Transporters (SWEETs) and Usually Multiple Acids Move In and out Transporters (UMAMIT) (Canarini et al. 2019). In *Arabidopsis* UMAMIT 14 and 18 have been associated with root secretion of amino acids (Besnard et al. 2016). This mechanism should allow for quick responses to local environment changes, such as biotic or abiotic stress responses. For example, Aluminum-activated Malate Transporters (ALMT) are upregulated under aluminum stress and phosphorous deficiency conditions (Canarini et al. 2019). This allows efflux of malate into the rhizosphere, which chelates to Aluminum (Al^3+^) and reduces toxicity (Jones 1998b; Canarini et al. 2019). From these examples, it is clear that passive transport processes play an important role in root-rhizosphere mediation.

#### Active transport

Active transporters can be of two varieties. The first is primary active, directly utilizing ATP to move compounds or protons against a concentration gradient (Canarini et al. 2019). The second form is secondary active antiport or symport, which does not directly utilize ATP but still relies upon an established concentration gradient to move two substrates together (Canarini et al. 2019). An example of this is MATE (Multidrug And Toxic compound Extrusion) transporters at the plasma membrane, which enable secretion of organic acids (Canarini et al. 2019). MATE/citrate transporters can be secondary antiporters, moving a proton down its concentration gradient while moving citrate against its concentration gradient (Canarini et al. 2019). There is research indicating an upregulation of active transport mechanisms under stress conditions for detoxification and mobilization of nutrient metals. For example, Pleiotropic Drug Resistance 1 (PDR1, an ATP-binding cassette/ABC transporter) is upregulated under phosphorous deficiency to increase strigolactone exudation for arbuscular mycorrhizal germination (Kretzschmar et al. 2012). Further active transport mechanisms must be in place for release of specialized metabolites, as unglycosylated specialized metabolites are predominantly secreted while their glycosylated counterparts remain within the cell (Farag et al. 2008).

We would propose that active transport mechanisms also play a role in border cell production processes. Release of border cells involves activation of pectin methylesterase, whose activity results in a decrease of the local root tip pH (Stephenson and Hawes 1994). A lower pH provides an ideal, proton rich environment for active movement of organic acids to the rhizosphere (i.e. a high proton concentration for utilization in active transport) with concurrent movement of protons into root cells. Rapid control of pH at the root tip region would also fit well into the overall context of border cell development and release. As aforementioned, border cells are rapidly replaced after their removal by water. In the event of water (of neutral or slightly basic pH) washing away border cells, there could be a concurrent increase in pH in the root tip local environment. An increase in pH (from slightly acidic to neutral or slightly basic) would provide a more favorable environment for pectin methylesterase activity, which has increased functionality from pH 5.1–7.4, with tenfold higher activity at pH 7.4 compared to pH 5.1 (Stephenson and Hawes 1994). The root apoplastic space is acidic (less than 6.4 in *Arabidopsis*) and the epidermal cell apoplastic pH changes depending on the pH of the surrounding environment (under low ionic medium conditions) (Martinière et al. 2018). As such, upon exposure to fluid that washes away border cells (such as water with a neutral pH around 7) there could be a considerable rise in the apoplastic pH in root epidermal tissues. This could initiate pectin methylesterase activity, resulting in new border cell production. As pectin methylesterase activity progresses, the root tip region would experience a decrease in pH, ultimately providing a proton rich environment for membrane transport activities and a lower pH for pectin methylesterase deactivation and cessation of border cell production.

#### Vesicular trafficking

Organic acids, amino acids, and sugars could also be released into the rhizosphere through exocytosis. This method of transport is seen in both border cells and root hairs (Ketelaar et al. 2008; Wang et al. 2017). As mentioned earlier in this review, border cells possess enhanced vesicular trafficking systems through which mucilage travels using large vesicles. This ultimately enables considerable mucilage rhizosphere deposition (Wang et al. 2017). This is an example of vesicular secretion, occurring directly from the trans-golgi network to the plasma membrane (Ekanayake et al. 2019). As mucilage can support microbial growth, this method of heavy secretion is important for its role in supporting the rhizosphere microbiome (Knee et al. 2001). Only border cells demonstrate this phenomenon, as root peripheral cells demonstrate some development of mucilage containing large vesicles, but they do not fuse to the plasma membrane until differentiating into border cells (Wang et al. 2017). This demonstration of such vesicular secretion in border cells presents possibilities for novel secretion mechanisms. However, the increased vacuolation and cytosolic vesicle presence within border cells indicates considerable reliance on vesicular trafficking to the vacuole (Wang et al. 2017; Ekanayake et al. 2019).

### Specialized metabolites and their roles in mediation of biotic and abiotic interactions

The remainder of this document will examine specialized/secondary metabolites and their origins within the various root tissues. Volatile compounds will be discussed first, as there is evidence supporting discrete tissue regions releasing these compounds. This section will conclude with discussion of non-volatile specialized metabolites.

#### Volatile compounds

The soil environment contains a plethora of volatile compounds, arising from plants, soil, and soil bound microbes (Ninkovic et al. 2019). Volatile compounds include terpenoids, fatty acid derivatives, phenylpropanoids, benzenoids, and amino acid derivatives (Qualley and Dudareva 2009; Dudareva et al. 2013). Some volatile compounds can be produced constitutively and are often referred to as phytoanticipins (Ninkovic et al. 2019). These compounds can also be conjugated to less volatile moieties to decrease overall volatility and prevent diffusion (Qualley and Dudareva 2009). Upon enzymatic cleavage of the sugar moiety, volatile compounds diffuse across plasma membranes readily, allowing for intracellular production and rapid movement to the extracellular/rhizosphere environment (Qualley and Dudareva 2009). Once released, volatile compounds can influence plant-plant, plant–microbe, plant–insect, and intracellular signaling processes. Because of the complexity of these interactions, there is still much work to be done sifting through the exact identities and functions of volatile compounds.

The roles and changes in plant volatiles can be divided into both biotic and abiotic responses. An example of a biotic response is aphid infestation, which results in plant release of airborne methyl-salicylate (Gong et al. 2023). This methyl-salicylate is perceived by neighboring plants via salicylic acid-binding protein 2 (SABP2), converted to salicylic acid, and initiates plant immune responses (Gong et al. 2023). Under abiotic stress conditions, volatile compounds emitted from plants change with increases in temperature, salt concentration, and light intensity (Ninkovic et al. 2019). Detected metabolites related to stress responses include isoprene, monoterpenes, sesquiterpenes, lipoxygenase products, and methanol (Ninkovic et al. 2019). These metabolites play a role in priming stress responses not just in the emitting-plants, but also in neighboring plants (Ninkovic et al. 2019). As volatile compounds travel quickly from their producing tissue, this would be an effective way to signal to other plants in the nearby region of a continuing or imminent stress. With volatile compounds priming stress responses in plants, future endeavors might explore leveraging the balance between higher constitutive volatile release compared to growth tradeoffs for agricultural applications.

While much work has been done examining overall root volatile releases under abiotic stress, studies examining the release of volatiles from discrete root tissues during abiotic stress are still limited. This lack of information is to be understood due to the complexity and challenges that come with examination of volatile compound release based on root tissue region under abiotic stress. However, increasing technological advancements in Solid Phase Micro-Extraction (SPME) coupled to Gas Chromatography-Mass Spectrometry (GC–MS) and its use in such studies is now within reach.

*M. truncatula* border cells have been shown to produce hexenal (also termed E-2-hexenal, 2-hexenal, or trans-2-hexenal, Pubchem 5,281,168), a compound associated with stress and plant response (Watson et al. 2015). While it appeared that this compound was secreted constitutively, it is possible collection of border cells resulted in some level of mechanical damage, defense activation and, as such, release of hexenal (Watson et al. 2015). Six carbon volatiles such as 2-hexenal are produced via hydroperoxide lyase enzymatic activity, which is a member of the lipoxygenase (LOX) pathway (Bate and Rothstein 1998). In coordination with this enzymatic pathway activity, qRT-PCR analysis of a LOX gene transcript that represents the branch point between jasmonate and hexenal biosynthesis indicated border cells have a 126-fold increase compared to the root tip (Watson et al. 2015). Furthermore, border cells possessed a fourfold increase in hydroperoxide lyase transcripts compared to the root tip, providing strong indication of border cell E-2-hexenal production (Watson et al. 2015). Emission of z-(3)-hexanal, a converted form of E-2-hexenal, from green leafy tissues primed defense responses in neighboring plants (Engelberth et al. 2004; Spyropoulou et al. 2017). In this particular study, the association was with prevention of insect herbivory in leaf tissues. However, it is possible that rhizosphere (i.e. root based) antimicrobial functions also exist. As hexenal has defense properties, the specific secretion of hexenal by border cells might be indicative of an underlying, and long posited, defense mechanism different than that of other tissues. In this mechanism, release of hexenal would aid in a form of “distraction” by attracting pathogenic bacteria. In *Arabidopsis*, application of E-2-hexenal resulted in increased growth of *Pseudomonas syringae* DC3000 (Scala et al. 2013). As such, it is possible that border cells are serving as decoys to attract microbial pathogens. Furthermore, since border cells are disposable/easily replaceable, serving as decoys to attract and neutralize pathogenic microbes (while protecting the critical root tip and meristem) aligns well with their known physiological functions. This would help prevent access to critical tissues underlying border cells (i.e. the root tip and meristem), allowing root growth to continue deeper into the soil away from danger. It is also possible that border cells release volatile compounds following exposure to microbial pathogens that could serve as a warning for the plant root. This would initiate a localized defense response, making border cells messengers to imminent infection. Supporting this theory, application of 10 µM trans-2-hexenal to *Arabidopsis* seedlings results in accumulation of anthocyanins (Bate and Rothstein 1998). Considering this evidence, a combination of functions, serving as both decoy and messenger, would make sense. Further supporting this possible function, combination of pea (*Pisum sativum* L.) border cells with *Nectria haematocca* (a fungal pathogen) resulted in quick border cell infiltration (Gunawardena and Hawes 2002). Removal of border cells revealed a low infiltration rate of the underlying root tip, with most infection occurring in border cells and the elongation zone (Gunawardena and Hawes 2002; Gunawardena et al. 2005). While border cell release of volatile compounds is promising, this specific activity is only a portion of the full bouquet and function of volatiles that can be released by plant roots (Huang et al. 2019). As such, there is considerable work to done in the realm of specific root tissue release of volatile compounds under biotic stress conditions. Again, it must be underscored that examination of volatiles from specific root tissues would be quite challenging, requiring specialized equipment and experimental methodologies.

#### Non-volatile specialized metabolites mediate symbiotic rhizosphere interactions

Root secreted specialized metabolites arise from the root tip, border cells, and root hairs (which are solely found on mature tissue). Legume root hairs, which are essential organs in formation of root nodules, secrete specialized metabolites in response to the localized presence of nodulating microbes (Brechenmacher et al. 2010). Successful metabolite-mediated communication results in symbiotic nodule formation and, ultimately, nitrogen fixation. More specifically, legume plant roots secrete phenolics, including flavonoids, which are nodulation inducing compounds recognized by rhizosphere microbes and initiate nodulation processes (Weston and Mathesius 2013). In nodulation, root hairs respond to soil microbial secreted lipochitooligosaccharide metabolites, which are often called nod factors and serve as part of the mutualistic signaling process (Bozsoki et al. 2017). Legumes possess receptors which enable root cells to differentiate the perception of chitin (an initiator of plant defense responses) from lipochitin oligosaccharides (Nod factors), which trigger endosymbiosis with rhizobial microbes (Bozsoki et al. 2017). While root hairs secrete initiators of nodulation, secretion of specialized metabolites seems to be primarily localized to the root tip region (Cesco et al. 2010). It should be mentioned that symbiotic interactions are not solely limited to nitrogen fixing bacteria, as select strains of arbuscular mycorrhiza promote growth in *Medicago truncatula* (Cope et al. 2022). In particular, strigolactones are secreted in response to phosphorous limiting conditions to attract arbuscular mycorrhizal fungi to help alleviate phosphorous deficiency (Cope et al. 2022).

The release of specialized metabolites is age-dependent, as new and developing tissues release high relative levels of specialized metabolites, with concentrations peaking at around 3 days in alfalfa seedlings (Maxwell et al. 1989; Cesco et al. 2010). As border cells are produced shortly after root emergence from seeds (within 5–10 mm root length), it is likely that much of the early specialized metabolite secretion arises from border cells (Hawes and Lin 1990; Stephenson and Hawes 1994). Logically speaking, the release of these metabolites early in root growth progress makes sense from two aspects. First and for legume species, the early release (i.e. seedling stage) of specialized metabolites translates to longer/earlier attraction of nodulating microbes. Effectively, a longer secretion period of symbiosis inducing metabolites can lead to higher concentrations, thus achieving the minimum concentration necessary for biological activity. Early secretion by border cells also recruits nodulating bacteria (specific metabolite identities discussed later in this section), which then would be present as the root continues to grow through that specific region of the rhizosphere. Thus, nodulating bacteria are present when the mature root region with root hairs eventually reaches the soil region favoring nodulation. This translates to greater fitness during plant maturation through enhancement of symbiotic interactions or direct specialized metabolite utilization to enhance nutrient acquisition/abiotic stress mediation. Secondly, many specialized metabolites have known antimicrobial functions (Zaynab et al. 2018). Early release of these antimicrobial compounds would also create a more favorable environment in soil regions by deterring pathogenic microbes. There is evidence that border cells specifically secrete antimicrobial metabolites (Watson et al. 2015; Kranawetter et al. 2021). There is evidence that these secretions could be leveraged or manipulated to produce high value compounds that increase plant fitness. An example of this is border cell production of *Cry*1Ac and *Cry*2Ab proteins from *Bacillus thuringiensis*, which is found in transgenic Bt-Cotton and helps with lepidopteran pest control (Knox et al. 2007). Border cells produced Cry protein at wide ranging levels, with estimates of 17–292% higher than that of root tips (Knox et al. 2007). While Cry proteins are not specialized metabolites, this evidence should encourage further study into manipulation of border cell secretions. Specific investigations into border cell contributions to the secreted specialized metabolite profile are ongoing.

Secreted specialized metabolites possess multiple roles in the rhizosphere. Some of these compounds serve dual functions in defense and symbiosis. For example, *Medicago sativum* (Alfalfa) and *Medicago truncatula* roots secrete 7,4’-dihydroxyflavone, an antifungal compound and initiator of nod genes in *Rhizobium meliloti* (which forms symbiotic nodules on Alfalfa) (Maxwell et al. 1989; Watson et al. 2015). Compounds chemically similar to 7,4′-dihydroxyflavone are stronger initiators of nodulation, such as apigenin, naringenin, hesperetin, luteolin (which is released from germinating seeds) and 4,4′-dihydroxy-2′-methoxychalcone (secreted by Alfalfa roots) (Maxwell et al. 1989; Begum et al. 2001; Liu and Murray 2016). Another chemically similar compound (7,4′-dihydroxyflavanone also known as liquiritigenin) is a weaker inducer of nod genes in *Rhizobium meliloti* in comparison to 7,4′-dihydroxyflavone (Maxwell et al. 1989). These examples demonstrate specificity in specialized metabolite structures involved with initiation of nodulation. However, it should be noted that they arise in multiple plant species, indicating possible broad utilization. For example, 4,4′-dihydroxy-2′-methoxychalcone is also released by the legume *Vicia sativa* and liquiritigenin is secreted by *Glycine max* (Soybean) (Liu and Murray 2016). Kaempferol, another root secreted specialized metabolite, possesses potent rhizosphere activity as an antimicrobial compound (Adamczak et al. 2020). This compound is secreted constitutively, potentially serving as a phytoanticipin (VanEtten et al. 1994). The exact origin of its secretion is unknown, although the enzyme-associated transcripts for kaempferol synthesis are upregulated in border cells, indicating a possible role of border cells in its secretion (Watson et al. 2015). Secretion of defense and symbiosis involved compounds creates a complicated network of communication/interactions in the rhizosphere.

Overall, plant secreted specialized metabolites allow rhizosphere microbiome manipulation, creating more favorable conditions for plant growth and development. The plant root specialized metabolite secreted profile changes, as expected and sometimes quite drastically, between individual plant species. For example, the rhizobia and nod factor induced legume root flavonoid secreted profile changes depending on plant species (e.g. *Glycine max*, *Phaseolus vulgaris*, *Medicago sativa*, *Vicia sativa*, *Trifolium subterraneum*, or *Pisum sativum*) (Subramanian et al. 2007; Liu and Murray 2016). Border cell secretions play a role in determination of host-microbe interactions, as their exudates activate bacterial reporter genes and nematode motility in a species-specific manner (Zhu et al. 1997; Zhao et al. 2000b). In effect, plant roots secrete a broad diversity of compounds that orchestrate specific soil microbiome modifications according to the needs of individual plant species.

## Root secretions in rhizosphere microbiome manipulation

Understanding the origin of rhizosphere secreted metabolites and their roles in the rhizosphere is becoming increasingly imperative. A growing body of literature has documented that plant roots modulate the rhizosphere microbiome, effectively selecting for certain microbial communities (Lebeis et al. 2015; Ma et al. 2021). For soil microbial communities, promotion of a healthy plant/root system enhances root exudation of carbon sources that plant growth promoting rhizobacteria (PGPR) can utilize (Mendes et al. 2013; Pieterse et al. 2014). Plants provided with ^13^CO_2_ (ultimately exuding ^13^C labeled compounds into the rhizosphere) demonstrated that root exudates are utilized by soil microbial communities (both bacterial and fungal) (Mendes et al. 2013). Rhizosphere bacteria congregate at root exudation regions, as the heaviest bacterial counts are seen around root tips and hairs (DeAngelis et al. 2009). Furthermore, select bacterial species are seen at higher relative counts in distinct root tissue regions (DeAngelis et al. 2009). For example, Bacteroidetes and Actinobacteria show decreased richness near the root tip compared to mature tissue regions (DeAngelis et al. 2009). However, observed increases/decreases in microbial richness depending on root tissue region would be expected to change based on host plant species as well as soil type, as these two factors have been shown to play a distinct role in soil microbiome diversity (Bulgarelli et al. 2013). Current work demonstrates that a variety of compounds can induce rhizosphere modulation, such as sugars, amino acids, and specialized metabolites (Liu and Murray 2016; Pini et al. 2017). An example of this is 7,4′-dihydroxyflavone, which increased the relative abundance of *Acidobacteria* subdivision 4 upon application to soil in a system modeling approximate exudation rates of *Medicago sativa* (Szoboszlay et al. 2016). It has also been demonstrated that 7,4′-dihydroxyflavone is at a relatively high concentration/intensity in both border cells and root secretions compared to the root tip, elongation zone, and mature tissue (Kranawetter et al. 2021). The biosynthesis and secretion of 7,4′-dihydroxyflavone appears to be primarily from border cells, as they have increased enzyme associated transcripts relative to all other root tissue types for Phenylalanine Ammonia Lyase (PAL, the first committed step in phenylpropanoid biosynthesis) and flavone synthase (the enzyme that forms 7,4′-dihydroxflavone from liquiritigenin) (Watson et al. 2015; Kranawetter et al. 2021). It should be noted that 7,4′-dihyroxyflavone is seen at a high relative intensity in the elongation zone as well, potentially indicating other root tissue types are involved in the secretion of this compound (Kranawetter et al. 2021).

The identification of metabolites responsible for rhizosphere microbiome manipulation is challenging, mostly because of the complexity of harvesting root exudates from soil for further analysis. The ability for plants to modify the soil microbiome and the soil microbiome to, in turn, modify plant growth should logically draw focus to further investigations on specific root-tissue secretory profiles. It is known that, holistically, plant root exudates influence the rhizosphere microbial community, assembling (or at minimum attempting to assemble) a microbial profile promoting growth. However, more specific work needs to be done to identify specific root regions (i.e. mature tissue, root tip, border cells, etc.) exuding bioactive compounds. Generalization of these tissues and their specific contributions to the overall root exudate profile neglects the power of differentiated root tissue types. Delving deeper into individual root tissue (i.e. mature tissue compared to border cells) contributions to the overall root exudate profile refines our knowledge of their underlying functions, thereby gaining deeper insight into the root-rhizosphere relationship.

More work is needed to incorporate metabolomics-based approaches to specific root segments and rhizosphere dynamics. Recent advances clearly indicate differential metabolite accumulation by root segment and a corresponding change in microbial populations (Loo et al. 2024). As future investigations delve deeper into understanding the selective impacts a given plant species has on its rhizosphere microbiome, attention should be paid to how specific, differentiated plant root tissue types contribute to rhizosphere manipulations. Extra attention should be paid to the relative localization of microbial communities based on specific root tissue regions. This will require root sectioning as part of future studies, with special consideration given to modern single cell sorting, sequencing, and metabolomics technologies.

## Concluding remarks

The current state of plant root research provides an evolving picture of metabolite secretions functioning in defense and commensal/symbiotic interactions. There is strong indication that bioactive metabolites arise from multiple tissue sources, with potential for inter-tissue communication. However, many studies still examine the root as a singular organ, neglecting the presence and role of differentiated tissues with unique functions. Future studies are encouraged to strive for higher spatial and tissue resolution and, importantly, with the inclusion of border cells. While existing work has demonstrated differential metabolite profiles based on individual root regions, more is needed to gain deeper understanding of the specific roles and functions of specialized root tissues and their role in the root-rhizosphere relationship (Moussaieff et al. 2013; Watson et al. 2015; Kranawetter et al. 2021). While defining metabolite profiles of specific root tissue types will certainly be complicated, current advancements of metabolomics technologies enable intricate studies using small amounts of tissue. As such, the major barrier remains tissue collection practices, which can be overcome using manual harvest with a scalpel, laser capture, or even fluorescence activated cell sorting (FACS). Advances in single cell metabolomics technologies should be followed diligently, as these will reduce the barrier to high-resolution metabolomics data by root segment. Furthermore, border cells should be included in future analyses investigating root-rhizosphere dynamics. A large body of literature shows border cells are complex players in root-rhizosphere interactions through secretion of bioactive metabolites (among other compounds). That being said, there is still a major knowledge gap in the isolated functions of border cells (i.e. what are they secreting and how) from an in-depth biochemical standpoint. Furthermore, there are few specifics on the response-based defense or symbiosis mechanisms that border cells employ. Further research in these two areas will establish a firm foundation on border cell functions in rhizosphere dynamics. From a long-term perspective, we must also delve deeper into identification of both border cell and individual root segment secretions across different species, which will shed light on the specifics underlying metabolite mediated plant–microbe interactions. With the availability of border cell and root segment collection strategies, along with the plethora of readily available, highly sensitive metabolomics instrumentation, there is great potential to delve deeper into the specific functions of individual root tissues.

## Data Availability

Not applicable.
